# Nuclear envelope structural defect underlies the main cause of aneuploidy in ovarian carcinogenesis

**DOI:** 10.1186/s12860-016-0114-8

**Published:** 2016-11-22

**Authors:** Callinice D. Capo-chichi, Toni M. Yeasky, Elizabeth R. Smith, Xiang-Xi Xu

**Affiliations:** 1 Sylvester Comprehensive Cancer Center/University of Miami, Miami, Florida 33136 USA; 2 Department of Cell Biology, University of Miami Miller School of Medicine, Miami, FL 33136 USA; 3 Institute of Biomedical Sciences, Laboratory of Biochemistry and Molecular Biology, University of Abomey-Calavi, Abomey Calavi, Benin

**Keywords:** Nuclear envelope, Lamin A/C, Aneuploidy, Polyploidy, Ovarian cancer, Carcinomas

## Abstract

**Background:**

The Cancer Atlas project has shown that p53 is the only commonly (96 %) mutated gene found in high-grade serous epithelial ovarian cancer, the major histological subtype. Another general genetic change is extensive aneuploidy caused by chromosomal numerical instability, which is thought to promote malignant transformation. Conventionally, aneuploidy is thought to be the result of mitotic errors and chromosomal nondisjunction during mitosis. Previously, we found that ovarian cancer cells often lost or reduced nuclear lamina proteins lamin A/C, and suppression of lamin A/C in cultured ovarian epithelial cells leads to aneuploidy. Following up, we investigated the mechanisms of lamin A/C-suppression in promoting aneuploidy and synergy with p53 inactivation.

**Results:**

We found that suppression of lamin A/C by siRNA in human ovarian surface epithelial cells led to frequent nuclear protrusions and formation of micronuclei. Lamin A/C-suppressed cells also often underwent mitotic failure and furrow regression to form tetraploid cells, which frequently underwent aberrant multiple polar mitosis to form aneuploid cells. In ovarian surface epithelial cells isolated from p53 null mice, transient suppression of lamin A/C produced massive aneuploidy with complex karyotypes, and the cells formed malignant tumors when implanted in mice.

**Conclusions:**

Based on the results, we conclude that a nuclear envelope structural defect, such as the loss or reduction of lamin A/C proteins, leads to aneuploidy by both the formation of tetraploid intermediates following mitotic failure, and the reduction of chromosome (s) following nuclear budding and subsequent loss of micronuclei. We suggest that the nuclear envelope defect, rather than chromosomal unequal distribution during cytokinesis, is the main cause of aneuploidy in ovarian cancer development.

## Background

Recently, the Cancer Atlas project [1] determined that mutation in p53 gene is the only common somatic genetic change (96 %) found in high-grade serous epithelial ovarian cancer [1, 2], the most common histological subtype. However, inactivation of p53 in ovarian epithelial cells in mouse models has not demonstrated a clear path for epithelial tumorigenesis [3, 4], even in aged mice following transplantation of p53 mutant ovaries [5]. Thus, the etiology and mechanism of epithelial ovarian cancer is not yet satisfactorily understood.

Another common genetic change in ovarian carcinomas revealed from the cancer genomic study is extensive aneuploidy [1]. The connection of abnormal chromosomes with cancer was first recognized over one hundred years ago by Boveri [6, 7]. Generally, aneuploidy is thought to be the result of mitotic errors and chromosomal nondisjunction during mitosis [8–10]. The majority of human ovarian cancer cells are aneuploid and possess a hyperdiploid (>46) to subtetraploid (<96) chromosome number [11]. Although a correlation between aneuploidy and malignancy has been recognized, the causes and significance of aneuploidy in cancer remain unsettled [7, 12–17]. Several mechanisms have been noted for the origination of aneuploidy [8, 10, 13, 18]. Genes that cause mitotic failure account for the majority of cases, and chromosomal non-disjunction is thought to cause unequal distribution of chromosomes in daughter cells [7, 10]. Tetraploid cells are believed to form following mitotic failure, and aneuploid cells are produced in subsequent mitotic events [8, 9, 13, 19]. Centrosome amplification also leads to multipolar cytokinesis and aneuploidy [18].

One unique view is that chromosome instability and aneuploidy may provide an unbalanced global expression profile of increases and decreases in gene dosages that create the cancer cell properties [12]. The general interpretation is that chromosome instability and aneuploidy promote the accelerated loss and gain of specific tumor suppressor genes and oncogenes, respectively, leading to selection of mutant cells with a growth advantage and subsequent malignant transformation [14, 16, 19, 20]. Two possible routes, a progressive shift up pathway and a tetraploid intermediate following drift down pathway, may convert a diploid normal cell to an aneuploid cancer cell. Cells with an optimal chromosome composition may have growth advantage, be selected, and become neoplastic.

Enlarged and deformed nuclei are characteristics of cancer cells, and the aberrant nuclear morphology correlates with malignancy and is a diagnostic and prognostic indicator, referred to as “nuclear grade” [21–26]. The increase in chromosome number over normal cells accounts for the larger nuclear size in cancer. Based purely on the nuclear morphology of cells sampled, the PAP test (or PAP smear), invented by Dr. Papanicolaou in the 1930s, is able to make diagnostic and prognostic prediction of the degree of malignancy of uterine and cervical cancers [27]. Changes in the nuclear matrix and/or nuclear envelope have been postulated, and deformation of nuclear morphology was shown to associate with oncogenic signaling [21, 28–31].

Shape of the nucleus is determined by structural proteins of the nuclear envelope lamina, which has been well studied [32–36]. Lamin A/C, but not lamin B1, is critical for the maintenance of a smooth and oval shaped nucleus [37]. Phosphorylation of lamin mediates reversible disassembly and re-formation of nuclear envelope in mitosis [38, 39]. Mutations or loss-of-function in several nuclear envelope structure proteins, including emerin, Man1, Baf, and lamin in *C. elegans*, cause similar nuclear and mitotic phenotypes such as an enlarged and deformed nucleus, defective chromosome segregation, and the formation of chromatin bridges between divided nuclei, suggesting a critical role for the nuclear envelope in cytokinesis and mitosis [33, 34, 38–41]. However, lamin A/C is dispensable for mitosis in mammalian cells since deletion of lamin A/C does not impair development in mice [42]. Nevertheless, lamin A/C is known to affect mitosis [43, 44], and a role in nuclear envelope formation likely influences the process of cytokinesis, though the redundancy of the three lamin genes present in mammals may reduce the impact of a single gene. Thus, roles of nuclear envelope proteins in maintaining the nuclear structure and mediating cytokinesis/mitosis are conserved across species. Lamin A/C expression is absent in embryonic stem cells and early embryos, and is progressively expressed in nearly all tissues in later developmental stages [45, 46]. The cell types that seem to lack lamin A/C, such as embryonic carcinoma cells and some cells of the spleen, thymus, bone marrow and intestine in the adult mouse, may fall into the “stem cell” category [45, 46].

Loss or reduction of lamin A/C expression is often found in cancer cells [47], including leukemia [48, 49], colon [50], prostate [29], lung [51], breast [52], and gastric cancers [53, 54]. Our earlier study also found that lamin A/C expression is lost in about 60 % of serous ovarian carcinomas, in which the mRNA is often present despite the loss of protein [55]. AKT and cell cycle associated phosphorylation of lamin A/C lead to this protein degradation [56–58]. One report concluded that lamin A/C proteins are increased in ovarian cancer when normal ovarian tissues (instead of ovarian surface epithelia) were used as controls [59]. However, the ovarian epithelial cells of the surface layer were found to be strongly positive, whereas the stromal cells were largely low for lamin A/C [59]. Thus, the correct interpretation of the result should be that 39 % of ovarian cancer cases are positive for lamin A/C, and lamin A/C proteins are lost or greatly reduced in 61 % of ovarian cancers. Another report identified lamin C as a marker that is reduced/lost in malignant ovarian cancer but not in borderline tumors based on results from 2-dimensional gel electrophoresis [60]. Thus, the published studies generally support our report that lamin A/C proteins are lost in over half of ovarian cancer. Previously, we found that suppression of lamin A/C caused aneuploidy in human ovarian surface epithelial cells [55]. Here, we further investigated the mechanisms and consequences of the development of aneuploidy and tumor development following the loss of lamin A/C using both human and mouse ovarian surface epithelial cells.

## Results

Previously using siRNA to suppress lamin A/C expression in human ovarian surface epithelial (HOSE) cells, we reported that loss of lamin A/C proteins led to a deformed nuclear morphology, polyploidy, and aneuploidy [55]. Following up the previous findings, we explored the mechanisms for the development of aneuploidy upon lamin A/C suppression. The HOSE cells were transfected with histone H2B-GFP that marks the nuclear DNA to monitor the behavior of the cells in cultures, as described previously [28]. Similar to that previously reported, about 50 % of HOSE cells expressed GFP after transfection, and the signals persisted for at the least 2 weeks over the length of the experiments. We observed that at any given time, 30 to 60 % of the lamin A/C-suppressed HOSE cells exhibited an aberrant nuclear morphology in about 200 cells analyzed. If allowed to follow the cells in cultures over several hours, essentially all cells displayed some degree of nuclear deformation, compared to about 5 % in controls that were transfected with scrambled siRNA oligonucleotides.

**Fig. 1:**
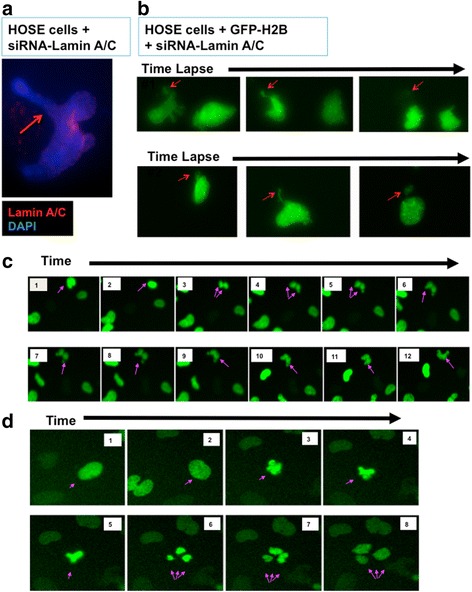
Nuclear protrusion and herniation, mitotic failure, and aberrant cytokinesis in lamin A/C-suppressed HOSE cells. Lamin A and C were suppressed by siRNA (Santa Cruz biotechnology Inc, Santa Cruz, CA) in primary human ovarian epithelial (HOSE) cells, using lipofectamine 2000 according to the manufacturer protocol (Invitrogen, CA). **a** At day 3, the lamin A/C-suppressed cells were analyzed using immunofluorescence microscopy. An example of a cell undergoing nuclear protrusion and herniation is shown (arrow), for nuclear staining with DAPI (*blue*) and lamin A/C (*red*). **b** The lamin A/C-suppressed primary HOSE cells that were previously transfected with GFP-histone H2B were monitored for nuclear changes by time-lapse video fluorescence microscopy over a 48-h period. Time-lapse video microscopy was performed 12 h after lamin A/C suppression. Cells were seeded in 24 well falcon plate and transfected the next day with scramble siRNA (control) or lamin A/C siRNA in serum reduced Opti-MEM media. Image acquisition was performed every 5 min for 48 h with a 40× dry objective lens on Nikon Eclipse TE 300 microscope linked to a Roper Scientific photometrics 12-bit range Camera using Meta imaging series (MetaVue) software. Stacked images were assembled with MetaVue software to make the movies. Two examples of sequential time-lapse images 15 min apart are shown. Arrows indicate the nuclear herniation and the formation of micronuclei, which gradually faded. Presumably, aneuploidy was resulted in the remaining nuclei. **c** Sequential time-lapse images (images frame #1 to 12) 15 min apart are shown for a 3-h segment of a video of a mitotic failure of a dividing cell. Arrows indicate the nuclei first underwent DNA condensation, separation, and then fused back to form presumably tetraploid nuclei. **d** Sequential time-lapse images (frame #1 to 8) 15 min apart show an aberrant mitotic process. Arrows indicate the nuclei undergoing a tripolar division. Likely, aneuploid cells were formed

One apparent feature of nuclear deformation was nuclear herniation, or budding (Fig. 1a, b). In around 20 % of over 200 cases examined, the nuclear body had an extremely extended herniation, and the nuclear materials often broke off to produce a micronucleus (Fig. 1a, b). When observed over a 6-h time-lapse, nearly all lamin A/C-suppressed cells underwent nuclear herniation and released micronuclei, as shown in two examples (Fig. 1b). Arrows indicate the nuclear protrusion and the formation of micronuclei, which gradually faded, presumably being degraded by the cellular proteolytic machinery. Consequently, aneuploid cells resulted, containing the remaining nuclei. In comparison, such phenomenon was rare in lamin A/C-positive HOSE cells, and about 4 % of over 200 control cells showed formation of micronuclei. Since lamin A/C is frequently reduced or lost in ovarian cancer cells [55], we reason that the lamin A/C-deficient ovarian cancer cells may develop aneuploidy by such a mechanism: nuclear protrusions and formation of micronuclei. The observations that cancer cell often undergo transient nuclear envelope rupture in gap phases [61] and collapse of micronuclei produced [62] support this idea.

Another feature of Lamin A/C-suppressed HOSE cells was frequent mitotic failure and the formation of tetraploid cells (Fig. 1c). In the analysis of 48-h time-lapse video, around a third (22 out of 80 mitotic events observed) of mitosis in GFP-histone H2B-labeled, siRNA-lamin A/C-transfected HOSE cells resulted in tetraploidy, as an example shown (Fig. 1c). Typically in such events, a cell was first observed to undergo nuclear condensation (Fig. 1c, frame #1–3) and subsequent attempt in cytokinesis (Fig. 1c, frame #4–8). However, the forming daughter nucleus failed to separate and then fused (Fig. 1c, frame #9–11), and a presumed tetraploid cell resulted (Fig. 1c, frame #12). In controls HOSE cells, mitotic failure was rare, none among 50 mitoses observed. In mammalian cells that have 3 lamin genes (lmna encoding lamin A/C, lmnb1, and lmnb2), lamin A/C is not essential for mitosis. However, lamin is essential for mitosis in C. elegans, which has only one lamin gene [43]. Nevertheless, mutations in lamin A/C interfere with mitosis and cell cycle progression in mammalian cells [43, 44]. Thus, our current finding that HOSE cells have an increased rate of mitotic failure seems compatible with the function of lamin proteins in cytokinesis during the formation of daughter nuclei in the dividing cells.

Additionally, aberrant mitosis such as tripolar cell division was frequent in lamin A/C-suppressed cells, as an example shown (Fig. 1d). The cells that underwent 3-way mitosis generally had larger nuclei, suggesting that they were tetrapoid cells, and thus aneuploid cells were generated. In the examination of 80 mitotic events of the lamin A/C-suppressed, GFP-Histone H2B-labeled HOSE cells, 6 events of aberrant tripolar mitosis were recognized.

The analysis of the cell behavior following lamin A/C suppression has been repeated over the course of 2 years, and the results were consistent from 4 independent preparations of HOSE cells. The above results led us to suggest that in lamin A/C-deficient cells, nuclear herniation to form micronuclei, mitotic failure to form tetraploid, and tripolar mitosis are mechanisms leading to the development of aneuploidy. Previously we found that lamin A/C-suppressed cells were not able to continue proliferation in culture, likely because of aneuploidy and activation of p53 check points [55].

**Fig. 2:**
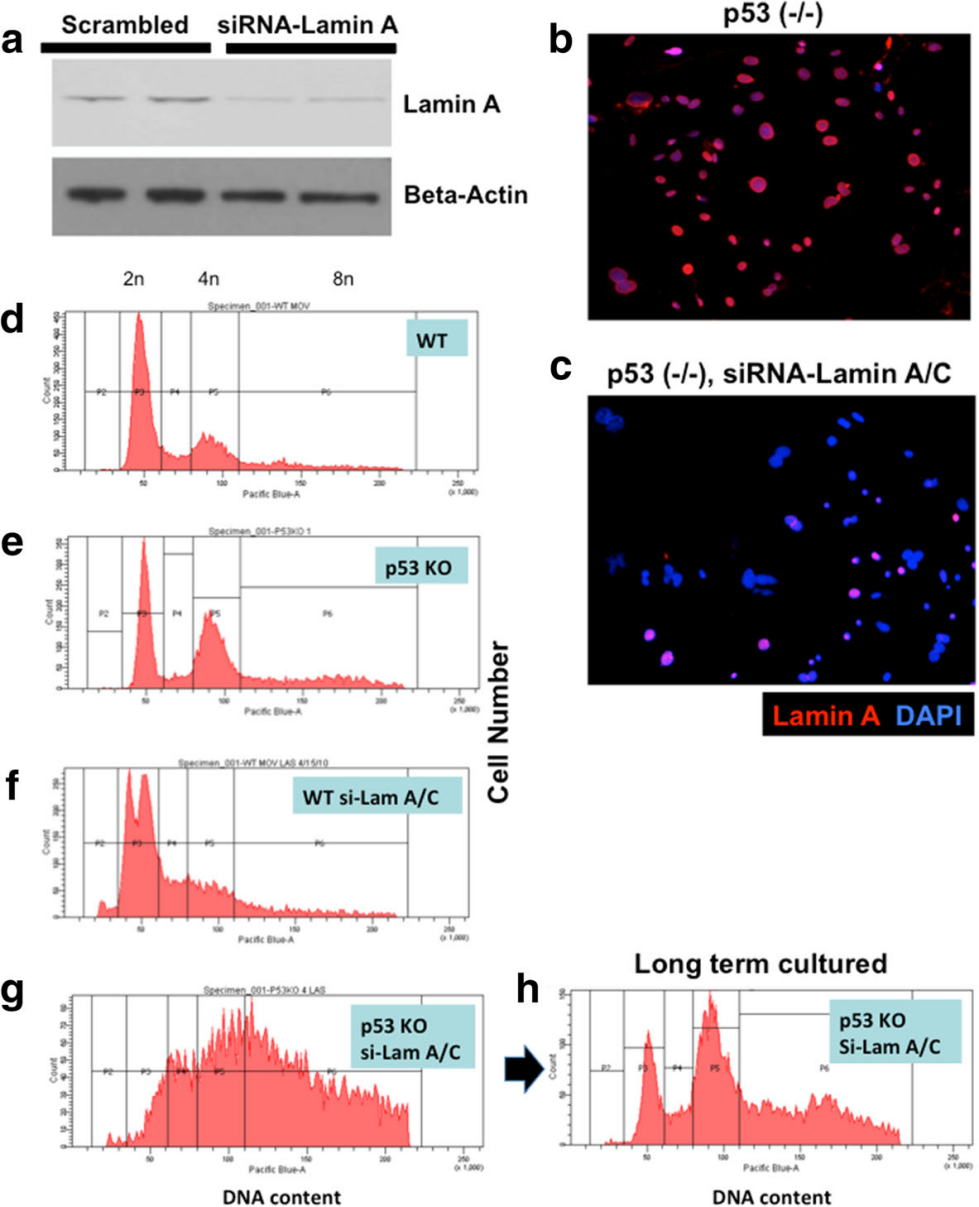
Lamin A/C suppression in primary mouse ovarian surface epithelial (MOSE) cells results in aneuploidy and polyploidy, synergistic with p53 deletion. Primary wildtype (WT) and p53 knockout (KO) MOSE cells were transfected with control (scrambled) or siRNA (si-Lam A) to suppress lamin A/C suppression. **a** At day 3, the lamin A/C-suppressed cells were analyzed by Western blot for the presence of lamin A protein. Duplicate experiments are shown for control (scrambled siRNA) and siRNA specific to mouse lamin A/C. **b** The cells were analyzed by immunofluorescence microscopy for the expression of lamin A. An example of p53 (-/-) MOSE cells treated with control siRNA is shown. **c** In comparison, staining was reduced in cells treated with siRNA-lamin A/C. **d** Flow cytometry was performed 3 days after siRNA transfection. Cells were collected following trypsin digestion, washed with PBS, and cell pellets were resuspended in ice cold ethanol/PBS (70 % v/v) with gentle agitation. The fixed cells were kept at − 20°C until ready to use. Prior to flow cytometric analysis, cells were centrifuged at 1200 rpm for 5 min and washed twice with PBS before resuspension in 0.5 mm vybrant *violet* dye. Cells were then incubated at 37°C for 30 min before flow cytometric analysis for DNA content. Flow cytometry profile for wildtype (WT) cells treated with control siRNA is shown. **e** p53 knockout cells; **f** WT cells treated with siRNA-lamin A/C; **g** p53 knockout cells treated with siRNA-lamin A/C. **h** Flow cytometry profile of the p53 knockout, siRNA-lamin A/C-treated MOSE cells following longer-term (2 months) culturing

We subsequently used primary mouse ovarian surface epithelial (MOSE) cells to further investigate the mechanism and consequence of lamin A/C-suppression. We reasoned that use of MOSE cells would allow us to introduce p53 genetic mutation to mimic the genotype in human ovarian cancer, and to bypass growth arrest following lamin A/C suppression. Primary ovarian surface epithelial cells were prepared from wildtype and p53 knockout mice, and were transfected with scrambled (control) or lamin A/C specific siRNA. The transfection efficiency ranged from 80 to 90 % in various experiments based on uptake of labeled cy3-siRNA oligonucleotides. Lamin A protein was significantly reduced upon transfection with targeting siRNA as visualized by Western blots (Fig. 2a). Using immunofluorescence microscopy, it was estimated that around 80 % of the MOSE cells had greatly reduced lamin A (Fig. 2b, c). Here, a lamin A - specific antibody was used, since it was found specific to the mouse protein, which is not properly recognized by several other available lamin A/C antibodies tested. The lamin A/C-suppressed MOSE cells also exhibited frequent nuclear herniation, mitotic failure, and aberrant mitosis (Fig. 2b, c), similar to those observed in HOSE cells upon suppression of lamin A/C expression.

We used flow cytometry to analyze cellular DNA content of the cells following siRNA suppression of lamin A/C. Comparing to the control cells (Fig. 2d) that have distinctive G1 (2n) and G2 (4n) peaks, p53 (-/-) MOSE cells showed a slightly higher fraction of polyploid (8n) cells (Fig. 2e). The lamin A/C-siRNA suppressed cells had a distinctive profile (Fig. 2f): the G1 peak separated into two (or more) main populations, which likely indicated the presence of a sub 2n fraction because of loss of one or few chromosomes by nuclear protrusion and the formation of micronuclei that was degraded. The G2 fraction was also reduced in lamin A/C-suppressed cells, likely because a cell cycle checkpoint was activated, as shown previously for HOSE cells [55]. In the p53 null and lamin A/C-suppressed cells, cell populations with various DNA content distributed continuously from 2n to 8n, suggesting the development of massive aneuploidy in these cells (Fig. 2g). Because of the presence of extensive aneuploidy, the profiles of these flow cytometry results were not suitable for analysis using a general flow cytometry program that does not account for aneuploidy.

Both the wildtype and the lamin A/C-suppressed MOSE cells had only limited life span in culture, and became senescent and deteriorated within 1–2 months. However, both the p53-deficient and the and the lamin A/C-suppressed p53-deficient MOSE cells continued to grow in culture. Following 4 weeks in culture, the original p53-deficient and Lamin A/C-suppressed MOSE cells with a wildly variable distributed chromosome number (Fig. 2g) converted into a more defined cellular chromosomal number distribution (Fig. 2h). We interpret that certain clones with optimal karyotypes from the original populations had growth advantage in culture and became the dominating cell populations.

**Fig. 3:**
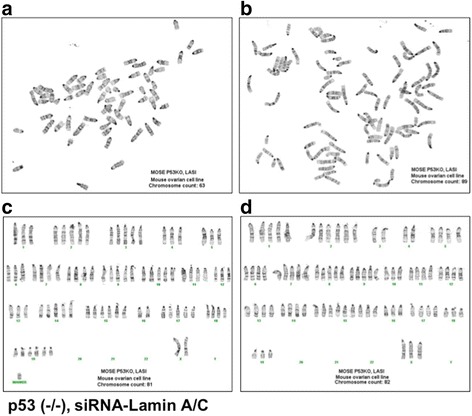
p53 inactivation and lamin A/C suppression result in aneuploidy and complex karyotypes. Primary p53 knockout MOSE cells were transfected with control or siRNA (si-Lam A) to suppress lamin A/C expression. The cells were maintained and passaged for 2 months in culture, and then subjected to chromosome analysis. Chromosome number counting and cytogenetic analysis were performed in 50 metaphase spreads for each cell preparation. At least 10 chromosome spreads from each preparation were randomly selected and estimated for chromosome number, and 2 appropriate samples were used for karyotyping. **a** and **b**, 2 representative examples of chromosome spreads from p53 (-/-) and siRNA-lamin A/C-treated MOSE cells are shown. **c** and **d**, 2 examples of karyotyping from p53 (-/-) and siRNA-lamin A/C-treated cells are shown

Indeed, chromosome analysis of metaphase spreads indicated aneuploidy and wide range of chromosomal number distribution in the lamin A/C-suppressed p53-deficient MOSE cells, such as 56, 60, 63, 67, 80, 81, 82, 84, 89, and 94 chromosomes, determined in 10 randomly selected metaphase spreads. Two of the examples are shown (Fig. 3a, b). Chromosome identification in two samples revealed complex karyotypes in the lamin A/C-suppressed p53-deficient MOSE cells (Fig. 3c, d), and a marker chromosome was observed in one sample (Fig. 3c). For comparison, metaphases from p53 knockout MOSE cells (without prior lamin A/C-siRNA treatment) were found to be largely near diploid (40 chromosomes) to tetraploid (80 chromosomes), and karyotyping by the cytogenetic core facility indicated that obvious structural abnormalities were not observed, but subtle abnormalities cannot be ruled out (quoted from the facility report).

**Fig. 4:**
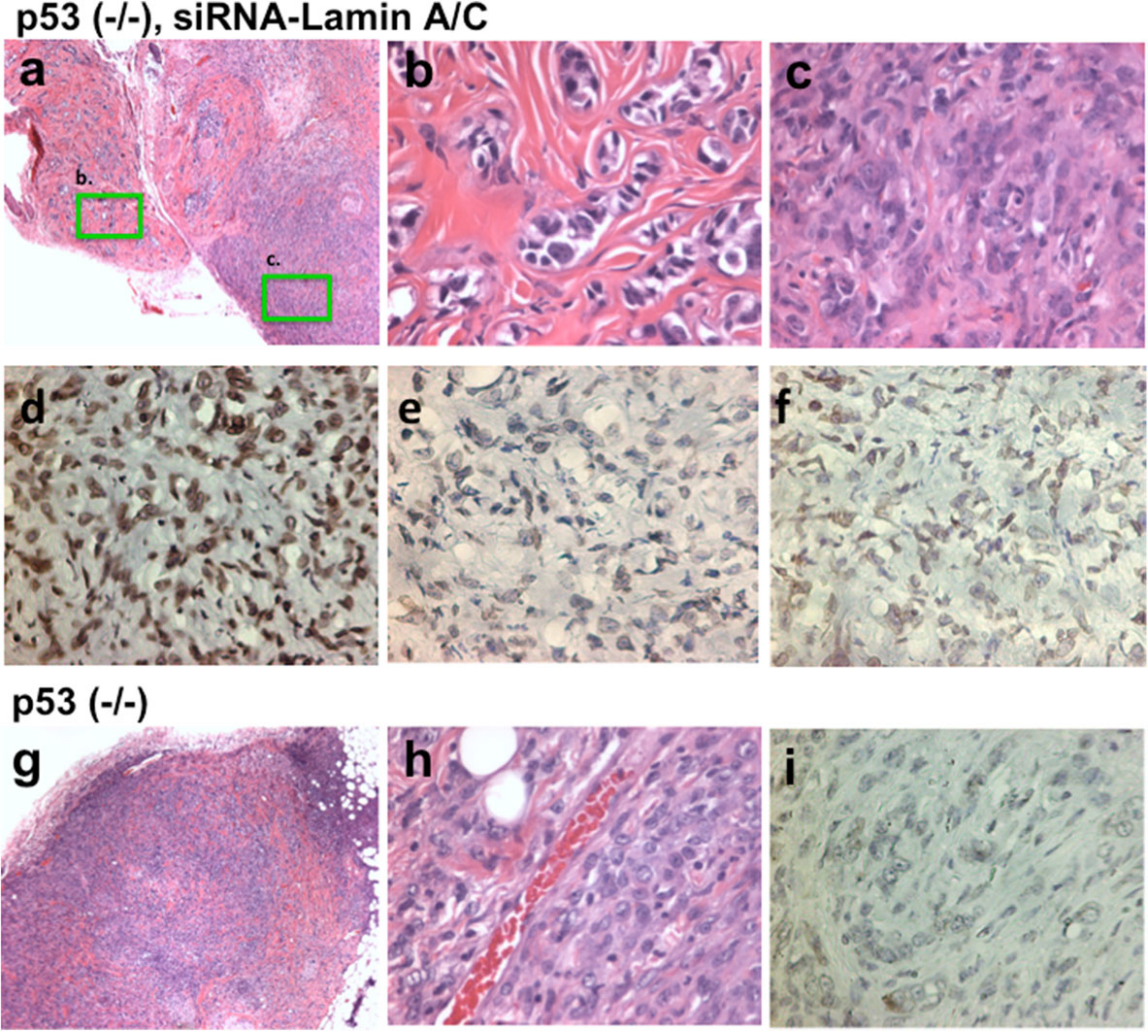
p53 inactivation and lamin A/C suppression lead to malignant tumors. Primary p53 knockout MOSE cells were transfected with control or siRNA (si-Lam A) and cultured for about 2 months. When the lamin A/C-suppressed and p53 (-/-) MOSE cells were implanted in nude mice subcutaneously, invasive tumors developed in 4 weeks (**a**). Two areas of the tumor are shown in higher magnification (**b**, **c**). Three examples of tumors formed from lamin A/C-suppressed p53 (-/-) MOSE cells were stained with lamin A/C, as shown in (**d**), (**e**), and (**f**). **g** Tumors formed from p53 (-/-) MOSE cells (not treated with lamin A/C-siRNA)) were compared, and a higher magnification (**h**) is shown. **i** An example of lamin A/C immunostaining is shown for a tumor derived for p53 (-/-) MOSE cells

When the MOSE cells were implanted into nude mice, both p53 (-/-) and lamin A/C-suppressed p53 (-/-) MOSE cells were tumorigenic (Fig. 4). Tumors formed in 5 of 6 nude mice when p53 (-/-) MOSE cells were implanted; and lamin A/C-suppressed p53 (-/-) MOSE cells formed tumors in 6 out of 6 mice tested. The tumors derived from the lamin A/C-suppressed p53 (-/-) MOSE cells had unique malignant features (Fig. 4a): the tumor cells often presented as small nodules invaded into muscle fibers (Fig. 4b, c). The tumor cells also exhibited a higher variation in nuclear sizes. In contrast, tumors derived from p53 (-/-) MOSE cells grew as a single mass with a more uniform nuclear morphology and size (Fig. 4g, h). Thus, a transient suppression of lamin A/C and generation of aneuploidy enable the growth of tumors with an increased degree of malignancy. Nevertheless, when implanted into immune competent female littermates from which the MOSE cells were prepared, neither p53 (-/-) nor lamin A/C-suppressed p53 (-/-) MOSE cells were able to produce significant or persistent tumors, indicating these MOSE cells were unable to escape the host immune surveillance in the development of tumors.

When the tumors were analyzed by immunohistochemistry, the tumors derived from lamin A/C-suppressed p53 (-/-) MOSE cells showed lamin A/C staining ranged from high (Fig. 4d), low (Fig. 4e), to mixed (Fig. 4f). Thus, it appears that some lamin A/C expression was recovered following the prior transc1ent suppression by siRNA. Interestingly, tumors derived from p53 (-/-) MOSE cells uniformly had low lamin A/C staining, in 3 out of 3 tumors analyzed as shown by a representative example (Fig. 4i). Thus, unexpectedly, the expression of lamin A/C is lower in tumors from p53 (-/-) MOSE cells without than with prior lamin A/C suppression. The results suggest a preference in growth and tumor development of a low laimin A/C cell population in the p53 (-/-) MOSE cells. The idea that a reduced lamin A/C expression may contribute to tumor development following p53 inactivation will need to be further verified.

## Discussion

Previously, we found that lamin A/C expression is commonly lost in ovarian cancer, and suppression of lamin A/C in ovarian surface epithelial cells led to the formation of aneuploidy, especially in p53 inactivated cells [55]. In following up the previous finding, here we determined the mechanisms for the development of aneuploidy when lamin A/C is eliminated. We found that when lamin A/C was suppressed, both the HOSE and the MOSE cells often failed in completing cytokinesis, and tetraploid cells were formed. Also, the aberrant nuclei underwent nuclear protrusion to form micronuclei, presumably a mechanism for progressive reduction of chromosome number to select for a growth permissive karyotype (s). Indeed, we observed that the lamin A/C-siRNA treated mouse p53 (-/-) MOSE cells exhibit complex karyotypes that resemble those of ovarian cancer cells.

The observation of frequent mitotic failure in lamin A/C-depleted cells is consistent with the requirement of lamin in cytokinesis in lower organisms such as *C. elegans* and *Drosophila* [33, 34], which have only one lamin gene. Possibly, the redundancy of the three lamin genes in mammals is the reason that the absence of a single lamin isoform would not generally cause cytokinesis failure, but rather increase the frequency of such events, as we have observed here.

**Fig. 5:**
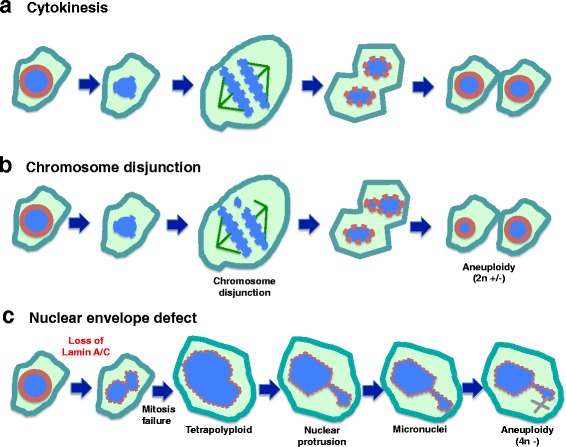
Working model: nuclear envelope defect is the main cause of aneuploidy in carcinogenesis. **a** Depiction of normal cytokinesis: at the start of M phase, the nuclear envelope dissolves, chromatin undergoes condensation, chromosomes pair and then separate, two new nuclear envelopes form, and cytokinesis is completed. **b** Chromosomal Disjunction: during chromosomal separation, one or more chromosomes are not attached. As a result, the two daughter cells have unequal distribution of chromosomes following cytokinesis. This mechanism is generally thought to be the main cause of aneuploidy. **c** Nuclear envelope defect causes aneuploidy: We reason that loss of a nuclear envelope structural component such as lamin A/C results in a misshapen nucleus. Additionally, the lamin A/C-deficient cells frequently fail to complete cytokinesis. Thus, tetraploid cells and subsequently aneuploid cells are generated. Formation of micronuclei at G-phases is another mechanism for the loss of individual chromosomes. Thus, we propose that the nuclear envelope defect is the main cause of aneuploidy in ovarian cancer development

The experimental results described here support a hypothesis that nuclear envelope defects (loss of lamin A/C proteins) may be the common cause of chromosomal numerical instability and aneuploidy in ovarian cancer (Fig. 5a-c). The idea explains both nuclear morphological deformation and aneuploidy, two prominent hallmarks of ovarian cancer. Generally, chromosomal disjunction is thought to be the cause of aneuploidy [7, 9, 10]. However, the results reported here leads us to a provocative hypothesis that nuclear envelope defect, such as loss of lamin A/C, rather than chromosomal disjunction (Fig. 5a), may be the main cause of aneuploidy in ovarian cancer (Fig. 5c). We reason that lamin A/C-deficient cells frequently fail to complete cytokinesis. We speculate that this is cause by the failure to properly form new nuclear envelope to encase the two new daughter nuclei, and the dividing nucleus undergoes furrow regression to produce tetraploid intermediates. Subsequently, aneuploid cells are generated by tripolar division. Formation of micronuclei at G-phases is another mechanism for the loss of individual chromosomes [61–64].

## Conclusions

Loss of lamin A/C appears to only increase the frequency of such mitotic failure in mammalian cells, though lamin is essential for cytokinesis in C. elegans, which has only one lamin gene [40]. Aneuploid cells may be growth retarded and undergo cell growth arrest or death [65, 66]. p53 mutation may allow the cells to survive and undergo clonal selection [67]. Most aneuploid cells generated from transient loss of lamin A/C likely would die, but ultimately, a population of cells with a unique chromosomal composition is selected and expanded to form cancer. Thus, our results advocate a concept that a deformed nuclear envelope is the main source of chromosomal instability of the cancer cells, and is the cause rather than a consequence of neoplastic transformation.

## Methods

### Reagents

Tissue culture flasks (Falcon), tissue culture media, trypsin, and 100× antibiotic-antimycotic solution (Cellgro, Mediatech, Inc) were purchased from Fisher Scientific Inc (Springfield, NJ). Triazol reagent and transfection reagent were purchased from Invitrogen Inc (Carlsbad, CA). For Western blot detection, Super Signal West Dura Extended Duration Substrate (PIERCE, Rockford, IL) was used. For immunofluorescence microscopy, Alexa Fluor 488 and 596 conjugated secondary antibodies and Hoechst 33342 nuclear counter staining dye were purchased from Molecular Probes Inc (Eugene, Oregon). Primary antibodies, anti-lamin A (H-102, rabbit polyclonal IgG), were purchased from Santa Cruz Biotechnology Inc (Santa Cruz, CA).

### Mouse models and xenograft tumor assay

The p53 knockout mice were purchased from Taconic (Hudson, NY) [68]. The mouse colony was kept in the C57BL/6 background and heterozygous pairs were bred to produce homozygous or wild type mice, which were used to prepare ovarian surface epithelial cells for experiments. The mouse colony was maintained and genotyped as described previously [69, 70].

Immunodeficient Scid mice were purchased from Jackson lab (Bar Harbor, ME). The mice were used for xenograft assays to test tumor development from MOSE cells by implanting 5 × 10^6^ cells subcutaneously in the immune deficient nude mice. The cells were tested to ensure free of microorganism contamination before xenograft assay. The mice were monitored daily post implantation to observe tumor development, up to 2 months. At the end of the experiments or when tumors with significant size (less than 10 % of body weight) were observed, the mice were euthanized and the tumors were dissected and subjected to histology analysis.

### Cell cultures

Primary human ovarian surface epithelial (HOSE) were isolated and provided by Dr. Andrew K. Godwin (Fox Chase Cancer Center). HOSE cells were cultured in media containing 6 g/l of HEPES, 15 % FBS, 1× antibiotic-antimycotic, and insulin, as reported previously [55]. Ovarian cancer cells were cultured in RPMI-1640 media supplemented with 10 % FBS and 1× antibiotic-antimycotic. All cells were maintained at 37 °C in a humidified atmosphere of 5 % CO_2_.

Primary mouse ovarian surface epithelial (MOSE) cells were isolated from 2 to 3 ovaries of p53 (-/-) or wild type BL6 mice of 3 to 6 months of age by collagenase digestion for an hour, as described previously [69, 70]. The cells released were harvested for culturing, and were found to be more than 90 % epithelial origin as characterized by cytokeratin staining [70]. These primary cells were used for experiments following a brief culture and expansion of 4 to 7 days.

### Small interfering RNA (siRNA) transfection

Lamin A/C expression in human cells (HOSE) was silenced using siRNA reagent (Catalog # sc-35776) purchased from Santa Cruz Biotech, Inc (Santa Cruz, CA). siRNA specific for mouse lamin A/C (Lmna (ID 16905) Trilencer-27 Mouse siRNA) was purchased from OriGene Inc. The siRNA is a mixture of 3 to 5 RNA oligonucleotides of 19–27 base long with sequences specifically targeting human lamin A/C, as described previously [55]. The siRNA oligonucleotides were transfected into HOSE or MOSE cells using Lipofectamine 2000 according to the manufacturer’s protocol (Invitrogen, CA). Cells were analyzed 72 h after transfection for Western blot and immunofluorescence microscopy analysis. Time-lapse video microscopy was performed 12 h after lamin A/C suppression. Cells were seeded in 24 well Falcon plate and transfected the next day with scrambled siRNA (control) or lamin A siRNA in serum reduced Opti-MEM media. The media was changed 10 h after transfection with phenol red free filming media containing 15 % FBS, HEPES, glutamine and antibiotic for ovarian epithelial cells. Time-lapse image acquisition was performed every 5 min for 48 h with a 40× dry objective lens on Nikon Eclipse TE 300 microscope linked to a Roper Scientific photometrics 12-bit range Camera. Image acquisition was done using Meta imaging series (MetaVue) software. Stacked images were assembled with MetaVue software to make the movie.

### Flow cytometric analysis

MOSE cells were seeded in T75 flasks and transfected the next day with siRNA in serum reduced Opti-MEM media. For fixed cells analysis, cells were re-suspended in ice-cold ethanol/PBS (70 % v/v) by gentle agitation. The fixed cells were kept at − 20 °C until assayed. Prior to flow cytometric analysis, cells were centrifuged at 1,200 rpm for 5 min and washed twice with PBS before re-suspension in 0.5 mm vybrant violet dye. Cells were then incubated at 37 °C for 30 min before flow cytometric analysis for DNA content, as described previously [28, 55].

### Immunofluorescence microscopy, time-lapse video, and immunohistochemistry

Briefly for immunofluorescence microscopy, adhered cells on 4-well chambered glasses were washed twice with PBS at room temperature, fixed with 4 % paraformaldehyde for 15 min, and permeablized with 0.5 % Triton X-100 for 5 min. Then, the cells were washed three times with PBS, blocked with 3 % BSA in PBS containing 0.1 % Tween-20 for 30 min, and incubated for 1 h at 37 °C with primary antibodies that were diluted (1/200) in 1 % BSA in PBS containing 0.1 % Tween- 20. AlexaFluor 488-conjugated (green fluorescence) or AlexaFluor 594-conjugated (red fluorescence) secondary antibodies were used. Nuclei and chromosomes were stained with Hoechst 33342 solution (1 M). Cells were washed three times, then mounted and sealed in anti-fade reagent containing 100 mM of n-propyl gallate (pH 7.4), 90 % glycerol in PBS. Immunofluorescence stainings were viewed with 60× or 100× oil objective lens on Nikon Eclipse TE 300 microscope linked to a Roper Scientific photometrics 12-bit range camera. Image acquisition was done using Meta imaging series (MetaVue) software. Images were merged using MetaVue software.

For time-lapse video microscopy, the acquisition of sequential image of cells expressing histone H2B-GFP was made every 5 min for up to 24 h as described previously [28].

### Karyotyping and chromosome analysis

Chromosome number counting and cytogenetic analysis were performed by the Cytogenetics & Molecular Diagnostic Laboratory of the University of Miami core facility. The cells were growth arrested at metaphase by incubation with colcemid for 8 h. For each cell preparation, 50 metaphase spreads were obtained and subjected to G-bands with 400 banding resolution. At least 10 chromosome spread samples from each preparation were randomly selected and estimated for chromosome number, and a few samples were used for chromosome identification by an experienced cytogenetist and certified by the facility director.

## Abbreviations

BSA: Bovine serum albumin
GFP: Green fluorescence protein
HOSE: Human ovarian surface epithelial
MOPS: 3-Morpholinopropane-1-sulfonic acid, 3-(N-Morpholino) propanesulfonic acid and 3-Morpholinopropanesulfonic acid
MOSE: Mouse ovarian surface epithelial
PBS: Phosphate-buffered saline
PMSF: Phenylmethylsulfonyl fluoride
SDS: Sodium dodecyl sulfate
siRNA: Small interfering RNA
TBS: Tris-buffered saline
TBST: Tris-buffered saline with Tween-20

## References

[1] Cancer Genome Atlas Research Network (2011). Integrated genomic analyses of ovarian carcinoma. Nature.

[2] Berchuck A, Kohler MF, Marks JR, Wiseman R, Boyd J, Bast RC (1994). The p53 tumor suppressor gene frequently is altered in gynecologic cancers. Am J Obstet Gynecol.

[3] Flesken-Nikitin A, Choi KC, Eng JP, Shmidt EN, Nikitin AY (2003). Induction of carcinogenesis by concurrent inactivation of p53 and Rb1 in the mouse ovarian surface epithelium. Cancer Res.

[4] Orsulic S, Li Y, Soslow RA, Vitale-Cross LA, Gutkind JS, Varmus HE. Induction of ovarian cancer by defined multiple genetic changes in a mouse model system. Cancer Cell. 2002;1(1):53–62. 10.1016/s1535-6108(01)00002-2 PMC2267863 12086888

[5] Chen CM, Chang JL, Behringer RR (2004). Tumor formation in p53 mutant ovaries transplanted into wild-type female hosts. Oncogene.

[6] Boveri T (1914). Zur Frage der Enstehung maligner Tumoren.

[7] Holland AJ, Cleveland DW (2009). Boveri revisited: chromosomal instability, aneuploidy and tumorigenesis. Nat Rev Mol Cell Biol.

[8] King RW (2008). When 2 + 2 = 5: the origins and fates of aneuploid and tetraploid cells. Biochim Biophys Acta.

[9] Shi Q, King RW (2005). Chromosome nondisjunction yields tetraploid rather than aneuploid cells in human cell lines. Nature.

[10] Jefford CE, Irminger-Finger I (2006). Mechanisms of chromosome instability in cancers. Crit Rev Oncol Hematol.

[11] Roschke AV, Tonon G, Gehlhaus KS, McTyre N, Bussey KJ, Lababidi S, Scudiero DA, Weinstein JN, Kirsch IR (2003). Karyotypic complexity of the NCI-60 drug-screening panel. Cancer Res.

[12] Duesberg P (2005). Does aneuploidy or mutation start cancer?. Science.

[13] Ganem NJ, Storchova Z, Pellman D (2007). Tetraploidy, aneuploidy and cancer. Curr Opin Genet Dev.

[14] Micho F, Iwasa Y, Vogelstein B, Lengauer C, Nowak MA (2005). Can chromosomal instability initiate tumorigenesis?. Semin Cancer Biol.

[15] Rajagopalan H, Lengauer C (2004). Aneuploidy and cancer. Nature.

[16] Storchova Z, Pellman D (2004). From polyploidy to aneuploidy, genome instability and cancer. Nat Rev Mol Cell Biol.

[17] Weaver BA, Cleveland DW (2006). Does aneuploidy cause cancer?. Curr Opin Cell Biol.

[18] Fukasawa K (2007). Oncogenes and tumour suppressors take on centrosomes. Nat Rev Cancer.

[19] Margolis RL (2005). Tetraploidy and tumor development. Cancer Cell.

[20] Pihan G, Doxsey SJ (2003). Mutations and aneuploidy: co-conspirators in cancer?. Cancer Cell.

[21] Boyd J, Pienta KJ, Getzenberg RH, Coffey DS, Barrett JC (1991). Preneoplastic alterations in nuclear morphology that accompany loss of tumor suppressor phenotype. J Natl Cancer Inst.

[22] Zink D, Fischer AH, Nickerson JA (2004). Nuclear structure in cancer cells. Nat Rev Cancer.

[23] Hsu CY, Kurman RJ, Vang R, Wang TL, Baak J, Shih IM (2005). Nuclear size distinguishes low- from high-grade ovarian serous carcinoma and predicts outcome. Hum Pathol.

[24] Palmer JE, Sant Cassia LJ, Irwin CJ, Morris AG, Rollason TP (2008). The prognostic value of nuclear morphometric analysis in serous ovarian carcinoma. Int J Gynecol Cancer.

[25] Partin AW, Walsh AC, Pitcock RV, Mohler JL, Epstein JI, Coffey DS (1989). A comparison of nuclear morphometry and Gleason grade as a predictor of prognosis in stage A2 prostate cancer: a critical analysis. J Urol.

[26] Pienta KJ, Coffey DS (1991). Correlation of nuclear morphometry with progression of breast cancer. Cancer.

[27] Papanicolaou GN (1942). A new procedure for staining vaginal smears. Science.

[28] Capo-chichi CD, Cai KQ, Testa JR, Godwin AK, Xu XX (2009). Loss of GATA6 leads to nuclear deformation and aneuploidy in ovarian cancer. Mol Cell Biol.

[29] Debes JD, Sebo TJ, Heemers HV, Kipp BR, Haugen DL, Lohse CM, Tindall DJ (2005). p300 modulates nuclear morphology in prostate cancer. Cancer Res.

[30] Fischer AH, Taysavang P, Jhiang SM (2003). Nuclear envelope irregularity is induced by RET/PTC during interphase. Am J Pathol.

[31] Nickerson JA (1998). Nuclear dreams: the malignant alteration of nuclear architecture. J Cell Biochem.

[32] Dechat T, Pfleghaar K, Sengupta K, Shimi T, Shumaker DK, Solimando L, Goldman RD (2008). Nuclear lamins: major factors in the structural organization and function of the nucleus and chromatin. Genes Dev.

[33] Gorjánácz M, Jaedicke A, Mattaj IW (2007). What can Caenorhabditis elegans tell us about the nuclear envelope?. FEBS Lett.

[34] Margalit A, Liu J, Fridkin A, Wilson KL, Gruenbaum Y (2005). A lamin-dependent pathway that regulates nuclear organization, cell cycle progression and germ cell development. Novartis Found Symp.

[35] Wilson KL (2000). The nuclear envelope, muscular dystrophy and gene expression. Trends Cell Biol.

[36] Wilson KL, Berk JM (2010). The nuclear envelope at a glance. J Cell Sci.

[37] Lammerding J, Fong LG, Ji JY, Reue K, Stewart CL, Young SG, Lee RT (2006). Lamins A and C but not lamin B1 regulate nuclear mechanics. J Biol Chem.

[38] Liu B, Wang J, Chan KM, Tjia WM, Deng W, Guan X, Huang JD, Li KM, Chau PY, Chen DJ, Pei D, Pendas AM, Cadiñanos J, López-Otín C, Tse HF, Hutchison C, Chen J, Cao Y, Cheah KS, Tryggvason K, Zhou Z (2005). Genomic instability in laminopathy-based premature aging. Nat Med.

[39] Heald R, McKeon F (1990). Mutations of phosphorylation sites in lamin A that prevent nuclear lamina disassembly in mitosis. Cell.

[40] Liu J, Rolef Ben-Shahar T, Riemer D, Treinin M, Spann P, Weber K, Fire A, Gruenbaum Y (2000). Essential roles for Caenorhabditis elegans lamin gene in nuclear organization, cell cycle progression, and spatial organization of nuclear pore complexes. Mol Biol Cell.

[41] Liu J, Lee KK, Segura-Totten M, Neufeld E, Wilson KL, Gruenbaum Y (2003). MAN1 and emerin have overlapping function (s) essential for chromosome segregation and cell division in Caenorhabditis elegans. Proc Natl Acad Sci U S A.

[42] Sullivan T, Escalante-Alcalde D, Bhatt H, Anver M, Bhat N, Nagashima K, Stewart CL, Burke B (1999). Loss of A-type lamin expression compromises nuclear envelope integrity leading to muscular dystrophy. J Cell Biol.

[43] Cao K, Capell BC, Erdos MR, Djabali K, Collins FS (2007). A lamin A protein isoform overexpressed in Hutchinson-Gilford progeria syndrome interferes with mitosis in progeria and normal cells. Proc Natl Acad Sci U S A.

[44] Dechat T, Shimi T, Adam SA, Rusinol AE, Andres DA, Spielmann HP, Sinensky MS, Goldman RD (2007). Alterations in mitosis and cell cycle progression caused by a mutant lamin A known to accelerate human aging. Proc Natl Acad Sci U S A.

[45] Lin F, Worman HJ (1997). Expression of nuclear lamins in human tissues and cancer cell lines and transcription from the promoters of the lamin A/C and B1 genes. Exp Cell Res.

[46] Röber RA, Weber K, Osborn M (1989). Differential timing of nuclear lamin A/C expression in the various organs of the mouse embryo and the young animal: a developmental study. Development.

[47] Foster CR, Przyborski SA, Wilson RG, Hutchison CJ (2010). Lamins as cancer biomarkers. Biochem Soc Trans.

[48] Agrelo R, Setien F, Espada J, Artiga MJ, Rodriguez M, Pérez-Rosado A, Sanchez-Aguilera A, Fraga MF, Piris MA, Esteller M (2005). Inactivation of the lamin A/C gene by CpG island promoter hypermethylation in hematologic malignancies, and its association with poor survival in nodal diffuse large B-cell lymphoma. J Clin Oncol.

[49] Stadelmann B, Khandjian E, Hirt A, Lüthy A, Weil R, Wagner HP (1990). Repression of nuclear lamin A and C gene expression in human acute lymphoblastic leukemia and non-Hodgkin’s lymphoma cells. Leuk Res.

[50] Willis ND, Cox TR, Rahman-Casañs SF, Smits K, Przyborski SA, van den Brandt P, Van Engeland M, Weijenberg M, Wilson RG, De Bruïne A, Hutchison CJ (2008). Lamin A/C is a risk biomarker in colorectal cancer. PLoS ONE.

[51] Machiels BM, Broers JL, Raymond Y, De Ley L, Kuijpers HJ, Caberg NE, Ramaekers FC (1995). Abnormal A-type lamin organization in a human lung carcinoma cell line. Eur J Cell Biol.

[52] Capo-chichi CD, Cai KQ, Smedberg J, Ganjei-Azar P, Godwin AK, Xu XX (2011). Loss of A-type lamin expression compromises nuclear envelope integrity in breast cancer. Chin J Cancer.

[53] Moss SF, Krivosheyev V, De Souza A, Chin K, Gaetz HP, Chaudhary N, Worman HJ, Holt PR (1999). Decreased and aberrant nuclear lamin expression in gastrointestinal tract neoplasms. Gut.

[54] Wu Z, Wu L, Weng D, Xu D, Geng J, Zhao F (2009). Reduced expression of lamin A/C correlates with poor histological differentiation and prognosis in primary gastric carcinoma. J Exp Clin Cancer Res.

[55] Capo-chichi CD, Cai KQ, Simpkins F, Ganjei-Azar P, Godwin AK, Xu XX (2011). Nuclear envelope structural defects cause chromosomal numerical instability and aneuploidy in ovarian cancer. BMC Med.

[56] Naeem AS, Zhu Y, Di WL, Marmiroli S, O’Shaughnessy RF (2015). AKT1-mediated Lamin A/C degradation is required for nuclear degradation and normal epidermal terminal differentiation. Cell Death Differ.

[57] Kochin V, Shimi T, Torvaldson E, Adam SA, Goldman A, Pack CG, Melo-Cardenas J, Imanishi SY, Goldman RD, Eriksson JE (2014). Interphase phosphorylation of lamin A. J Cell Sci.

[58] Bertacchini J, Beretti F, Cenni V, Guida M, Gibellini F, Mediani L, Marin O, Maraldi NM, De Pol A, Lattanzi G, Cocco L, Marmiroli S (2013). The protein kinase Akt/PKB regulates both prelamin A degradation and Lmna gene expression. FASEB J.

[59] Hudson ME, Pozdnyakova I, Haines K, Mor G, Snyder M (2007). Identification of differentially expressed proteins in ovarian cancer using high-density protein microarrays. Proc Natl Acad Sci U S A.

[60] Alaiya AA, Franzén B, Fujioka K, Moberger B, Schedvins K, Silfversvärd C, Linder S, Auer G (1997). Phenotypic analysis of ovarian carcinoma: polypeptide expression in benign, borderline and malignant tumors. Int J Cancer.

[61] Vargas JD, Hatch EM, Anderson DJ, Hetzer MW (2012). Transient nuclear envelope rupturing during interphase in human cancer cells. Nucleus.

[62] Hatch EM, Fischer AH, Deerinck TJ, Hetzer MW (2013). Catastrophic nuclear envelope collapse in cancer cell micronuclei. Cell.

[63] Aristei C, Stracci F, Guerrieri P, Anselmo P, Armellini R, Rulli A, Barberini F, Latini P, Menghini AR (2009). Frequency of sister chromatid exchanges and micronuclei monitored over time in patients with early-stage breast cancer: results of an observational study. Cancer Genet Cytogenet.

[64] Shimizu N, Itoh N, Utiyama H, Wahl GM (1998). Selective entrapment of extrachromosomally amplified DNA by nuclear budding and micronucleation during S phase. J Cell Biol.

[65] Torres EM, Williams BR, Amon A (2008). Aneuploidy: cells losing their balance. Genetics.

[66] Williams BR, Prabhu VR, Hunter KE, Glazier CM, Whittaker CA, Housman DE, Amon A (2008). Aneuploidy affects proliferation and spontaneous immortalization in mammalian cells. Science.

[67] Thompson SL, Compton DA (2010). Proliferation of aneuploid human cells is limited by a p53-dependent mechanism. J Cell Biol.

[68] Jacks T, Remington L, Williams BO, Schmitt EM, Halachmi S, Bronson RT, Weinberg RA (1994). Tumor spectrum analysis in p53-mutant mice. Curr Biol.

[69] Cai KQ, Wang Y, Smith ER, Smedberg JL, Yang DH, Yang WL, Xu XX (2015). Global deletion of Trp53 reverts ovarian tumor phenotype of the germ cell-deficient white spotting variant (Wv) mice. Neoplasia.

[70] Wang Y, Cai KQ, Smith ER, Yeasky TM, Moore R, Ganjei-Azar P, Klein-Szanto AJ, Godwin AK, Hamilton TC, Xu XX (2016). Follicle Depletion Provides a Permissive Environment for Ovarian Carcinogenesis. Mol Cell Biol.

