# Erratum to: Nuclear envelope structural defect underlies the main cause of aneuploidy in ovarian carcinogenesis

**DOI:** 10.1186/s12860-016-0124-6

**Published:** 2017-01-09

**Authors:** Callinice D. Capo-chichi, Toni M. Yeasky, Elizabeth R. Smith, Xiang-Xi Xu

**Affiliations:** 1 Sylvester Comprehensive Cancer Center/University of Miami, Miami, Florida 33136 USA; 2 Department of Cell Biology, University of Miami Miller School of Medicine, Miami, FL 33136 USA; 3 Institute of Biomedical Sciences, Laboratory of Biochemistry and Molecular Biology, University of Abomey-Calavi, Abomey Calavi, Benin

## Erratum

This article [1] contains an updated version of Fig. 5. The original version has been corrected due to errors introduced during the process of illustration. The updated figure is also shown on the following page:

**Fig. 5:**
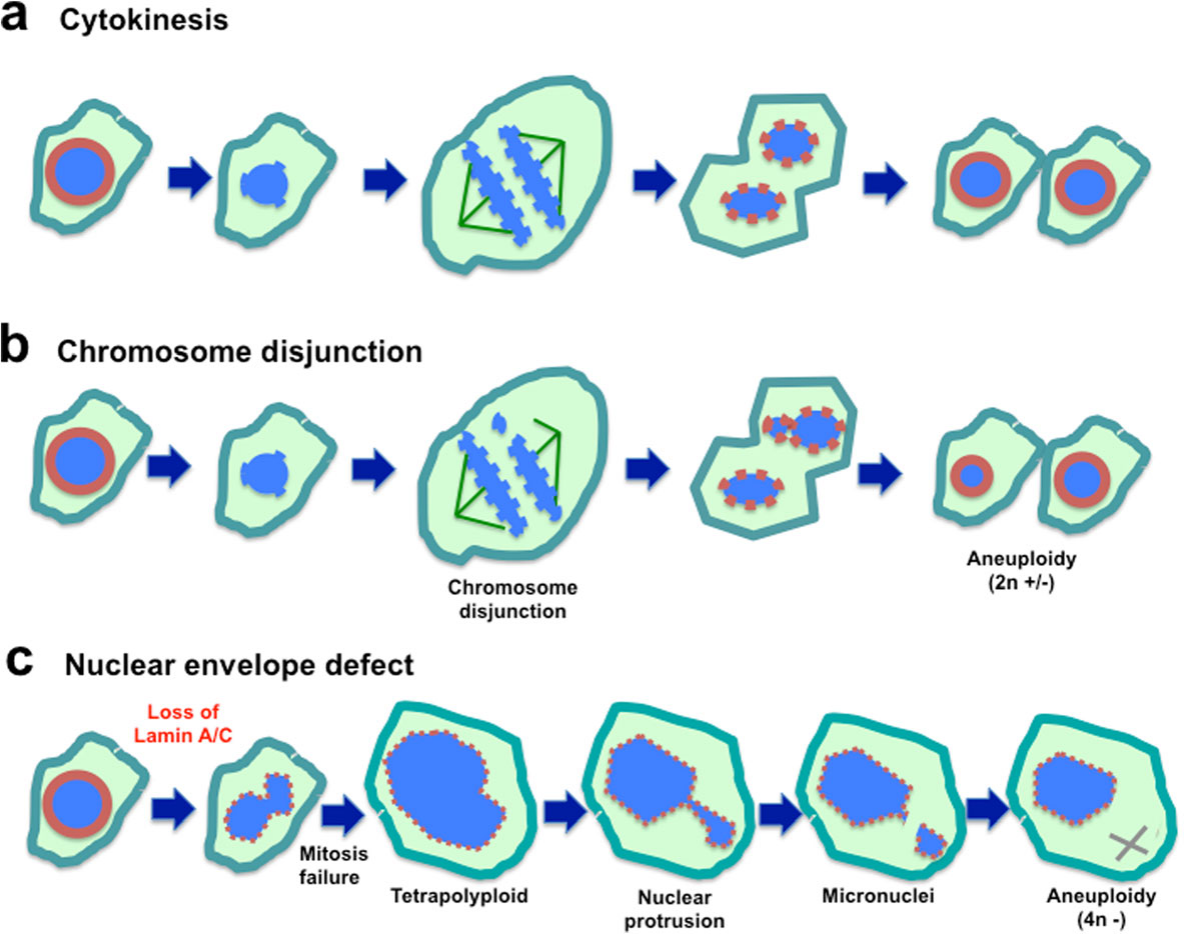
Working model: nuclear envelope defect is the main cause of aneuploidy in carcinogenesis. **a** Depiction of normal cytokinesis: at the start of M phase, the nuclear envelope dissolves, chromatin undergoes condensation, chromosomes pair and then separate, two new nuclear envelopes form, and cytokinesis is completed. **b** Chromosomal Disjunction: during chromosomal separation, one or more chromosomes are not attached. As a result, the two daughter cells have unequal distribution of chromosomes following cytokinesis. This mechanism is generally thought to be the main cause of aneuploidy. **c** Nuclear envelope defect causes aneuploidy: We reason that loss of a nuclear envelope structural component such as lamin A/C results in a misshapen nucleus. Additionally, the lamin A/C-deficient cells frequently fail to complete cytokinesis. Thus, tetraploid cells and subsequently aneuploid cells are generated. Formation of micronuclei at G-phases is another mechanism for the loss of individual chromosomes. Thus, we propose that the nuclear envelope defect is the main cause of aneuploidy in ovarian cancer development
